# Genomic prediction models for grain yield of spring bread wheat in diverse agro-ecological zones

**DOI:** 10.1038/srep27312

**Published:** 2016-06-17

**Authors:** C. Saint Pierre, J. Burgueño, J. Crossa, G. Fuentes Dávila, P. Figueroa López, E. Solís Moya, J. Ireta Moreno, V. M. Hernández Muela, V. M. Zamora Villa, P. Vikram, K. Mathews, C. Sansaloni, D. Sehgal, D. Jarquin, P. Wenzl, Sukhwinder Singh

**Affiliations:** 1 International Maize and Wheat Improvement Center (CIMMYT), Km. 45, Carretera México-Veracruz, El Batán, Texcoco, CP 56237, México; 2 Instituto Nacional de Investigaciones Forestales, Agrícolas y Pecuarias, INIFAP, México; 3 Universidad Autónoma Agraria Antonio Narro, México; 4 Department of Agronomy and Horticulture, University of Nebraska-Lincoln, 321 Keim Hall, Lincoln, NE, 68503-0915, USA

## Abstract

Genomic and pedigree predictions for grain yield and agronomic traits were carried out using high density molecular data on a set of 803 spring wheat lines that were evaluated in 5 sites characterized by several environmental co-variables. Seven statistical models were tested using two random cross-validations schemes. Two other prediction problems were studied, namely predicting the lines’ performance at one site with another (pairwise-site) and at untested sites (leave-one-site-out). Grain yield ranged from 3.7 to 9.0 t ha^−1^ across sites. The best predictability was observed when genotypic and pedigree data were included in the models and their interaction with sites and the environmental co-variables. The leave-one-site-out increased average prediction accuracy over pairwise-site for all the traits, specifically from 0.27 to 0.36 for grain yield. Days to anthesis, maturity, and plant height predictions had high heritability and gave the highest accuracy for prediction models. Genomic and pedigree models coupled with environmental co-variables gave high prediction accuracy due to high genetic correlation between sites. This study provides an example of model prediction considering climate data along-with genomic and pedigree information. Such comprehensive models can be used to achieve rapid enhancement of wheat yield enhancement in current and future climate change scenario.

Global wheat production is currently close to 700 million tons^1^, and the demand for wheat in developing countries is projected to increase 60% by 2050^2^. Wheat grain yield is a complex trait that depends on multiple genes interacting with each other and the environment^3^,^4^. Although the effects of major genes regulating plant phenology and morphology and their influence on grain yield have been previously described^5^, quantitative trait loci (QTLs) for grain yield have had limited practical applications in breeding programs due to the small genetic variance accounted for by individual QTLs, the variation across environments^4^, and the influence of the genetic backgrounds.

Recent advances in sequencing technologies have enabled the generation of high throughput, fast, and relatively inexpensive genotypic information; thereby facilitating the implementation of genomic prediction and genomic selection in plant and animal breeding^6^. Incorporation of genomic information through prediction models provides an alternative approach to indirect selection in breeding for crop varieties. Given that plant breeding programs started to incorporate genomic information, parametric linear regression and non-parametric models have emerged as preferred methods^7^,^8^. However, the genetic instruction from genes translates into the full set of phenotypic traits and ultimately into grain yield components is affected by numerous interactions among pathways and the environment. Genotype by environment interactions (G × E) can reduce trait heritability and the ability to statistically predict superior genotypes under contrasting environments^9^,^10^. For this reason, collecting phenotypic data from different environments continues to be a powerful predictor of important biological outcomes such as grain yield^11^. Although different genomic technologies are being utilized to breed suitable varieties, genomic selection provides the option of considering multiple variables simultaneously for predicting genetic yield potential^10^.

Pedigree information accounts for the proportion of predictive ability related to differences in families and increases prediction accuracy when used together with marker information (that accounts for Mendelian sampling) in genomic selection models^12^. Burgueño *et al.*^9^ demonstrated the superiority of pedigree plus genomic models over pedigree or genomic-based predictions alone when incorporating G × E in the genomic regression model. Jarquin *et al*.^13^ proposed a model that can use not only genomic information but also pedigree and environmental information for the prediction of unobserved genotypes. Data from multi-environment trials can also be used for predicting climate change scenarios and selecting suitable sites for evaluating promising germplasm. Including environmental covariables in genomic selection prediction models is expected to result in less biased estimation of effects, higher prediction accuracy, better precision and power, and increased heritability to explain grain yield variation^14^. This information facilitate selection of promising germplasm for use in crop breeding aimed at both population improvement and cultivar release.

Cross-validation schemes are used in genomic prediction studies to estimate the accuracy with which predictions can be made for different traits and environments^9^,^15^,^16^,^17^,^18^,^19^,^20^,^21^,^22^. There are two basic cross-validation schemes used in genome-enabled prediction: (1) predicting the performance of certain proportion of lines that have not been evaluated in any of the observed environments (CV1), and (2) predicting the performance of a proportion of lines that have been evaluated in some environments, but not in others, also called sparse testing (CV2). Another prediction problem that does not involve random cross-validation is predicting one environment using another environment (pairwise environment). The fourth prediction problem consists of predicting one environment (i.e., site-year combination) that was not included in the usual set of testing environments in the evaluation system (leave-one-environment-out); the only available information on this untested environment could be certain characteristics that would have been previously collected such as soil type, altitude, longitude, maximum and minimum temperature, precipitation during other cropping cycles, etc. It is expected that predicting the performance of untested lines can be conducted with sufficient accuracy when there is knowledge about their relationships (pedigree relationship or genomic relationship). Similarly, the performance of lines in unobserved environments could be predicted if there is information about the environmental conditions^17^. The accuracy of predicting performance in unobserved environments would however be related to our ability to select the most appropriate environmental variables for inclusion in the prediction model. To date, this would be the first study assessing the prediction problems when leaving-one-environment-out with real environmental data.

In light of the facts mentioned above, the following objectives of the present study were framed: 1) to investigate the stability performance of wheat lines across a set of 5 Mexican environments; 2) to evaluate genomic prediction with high density genotype-by-sequencing (DArTseq) markers for agronomic traits and grain yield using different combinations for the effects of lines (L), sites (E), genomic data (G), pedigree data (A), and environmental covariables (W) and their interactions; and 3) to test a new problem that arises when predicting the performance of wheat lines in environments that have not been previously used (untested environments) where the only available information from them is their climate data.

## Results

### Genetic variance of site and genotypic correlation between sites

High genetic correlations were observed among sites for days to anthesis, days to maturity, plant height, and for grain yield at most of the pairwise sites (Table 1). Broad sense heritability for plant height, days to anthesis, and maturity in all the environments, was relatively higher than that of grain yield, except in Celaya (data not presented).

### Phenotypic variability of the traits measured across sites

**Figure 1:**
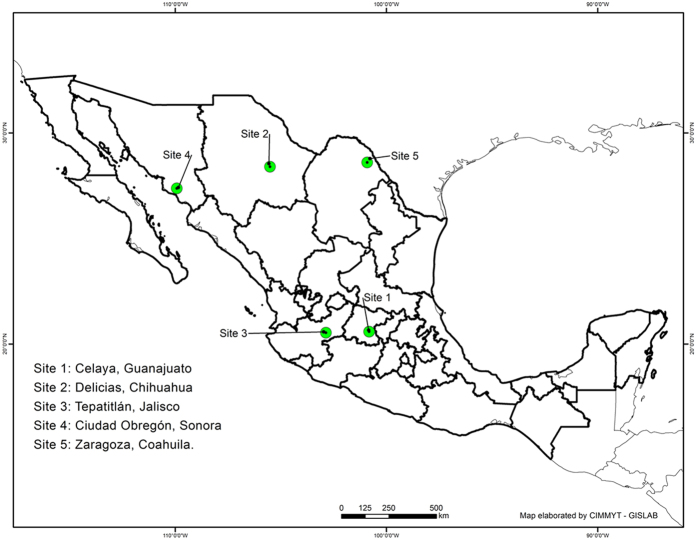
Geographic distribution of the five sites where the field trials were conducted. Map was constructed using ESRI’s ArcGIS Desktop ArcMap 10.2.2 software (26).

**Figure 2:**
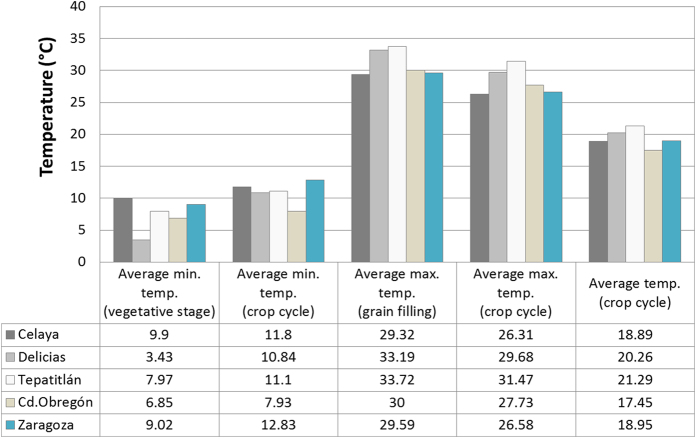
Environmental co-variables used to build the W matrix, including i) Average minimum temperature at vegetative stage, ii) Average minimum temperature during crop cycle, iii) Average maximum temperature during grain filling, iv) Average maximum temperature during crop cycle, and v) Average temperature during crop cycle. Sites refer to Celaya; Delicias; Tepatitlán; Ciudad Obregón and Zaragoza.

**Figure 3:**
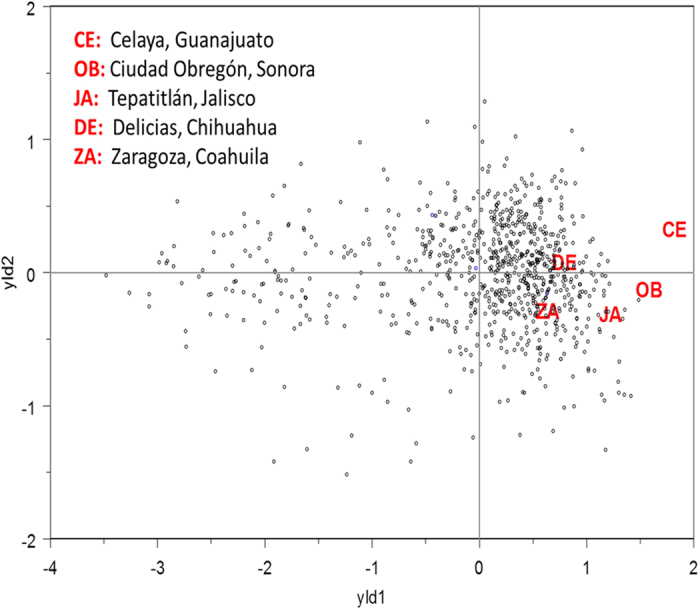
Bi-plot for grain yield. The red codes represent each of the sites. The points represent each of the lines. The lines closest to the end of the site vector are the best performing lines for that specific site.

Sites represented different wheat growing conditions in Mexico, from 39 meters masl (Cd. Obregón) to 1,930 masl (Tepatitlán) and differences in latitude of 8 degrees (Fig. 1, Supplementary Table 1). Average minimum, mean and maximum temperatures during different critical phases of the crop cycle are presented in Fig. 2. Celaya was the warmest site during the first stage of the crop and one of the coldest during grain filling. Conversely, Delicias was the coldest site during the vegetative stage and the warmest site during the grain filling period. Mean grain yield varied significantly across environments, ranging from 3.7 t ha^−1^ at Zaragoza to a maximum of 9 t ha^−1^ at Tepatitlán. For grain yield, a combined analysis of genetic correlations and genetic variances revealed positive genetic correlations between sites, as shown in the bi-plot (Fig. 3). Figure 3 revealed clustering of sites into one group. Similarly, positive genetic correlations were illustrated by the vectors from the origin of graph to the sites (letters), when ranking genotypes on grain yield. The separation of the sites (see letters in the graph) from the origin (center of the graph) is an indicator of the higher heritability values for those sites and thus, a measure of the site’s effective discriminating power. Celaya and Cd. Obregón showed the maximum separation from the origin and, were therefore, the most effective sites for identifying genetic differences between genotypes. It is important to note that the temperature regimes in Celaya and Cd. Obregón were similar during anthesis, as shown in Fig. 2.

Genotypes do not show any clear pattern in the bi-plot for grain yield (Fig. 3). Most of the genotypes are located in a cloud at a value of zero and right in the first dimension and a left tail of genotypes. Similar results were found for the other traits analyzed (bi-plot not presented).

**Figure 4:**
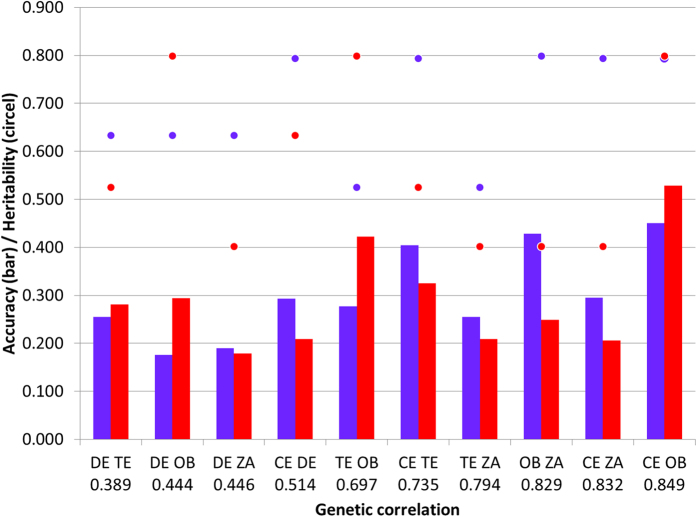
Bar plot of the prediction accuracy for all pairwise-site arranged by their genetic correlations for grain yield. The heritability for each site within the pair is represented by a circle. For each pair-wise the prediction is direct and reciprocal. Abbreviations: CE: Celaya; DE: Delicias; TE: Tepatitlán; OB: Cd. Obregón; ZA: Zaragoza.

For grain yield the sites were intermediately to highly genetically correlated (0.4**–**0.85) (Table 1). The correlations between the pair of sites are related to the prediction accuracy for each pairwise-site correlation as depicted in Fig. 4. For example for the pair of sites with the highest correlation (0.85), Celaya predicts Cd. Obregón well but Cd. Obregón predicts Celaya with slightly less accuracy. Furthermore, Cd. Obregón and Zaragoza had a genetic correlation of 0.829 and Zaragoza predicts Cd. Obregón well, but not vice versa.

### Genomic prediction analysis for grain yield and phenology

Among the seven tested prediction models for grain yield, the models E+W+G+A and E+W+G+A+GE+AE performed better than other models in cross validation schemes CV1 and CV2, respectively (Table 2). The highest correlation value in CV1 and CV2 was obtained in Cd. Obregon, followed by Celaya. Though not absolutely, these two models (E+W+G+A and E+W+G+A+GE+AE) performed better than other models for highly heritable traits, i.e., days to anthesis and days to maturity and plant height (Supplementary Table 2). In terms of sites, comparatively better predictions were observed when Celaya was used as a training set to predict Cd. Obregón (Table 3). Results clearly revealed that after including the **G** (genomic data) or **A** (pedigree information) matrix in the model, prediction ability increased.

Pairwise-site prediction accuracy for grain yield is shown in Table 3 with a noticeable increase at most the sites in the prediction accuracy of models E+W+A and E+W+G+A over the other two models. Model E+W+A was the best model when Celaya, Delicias and Tepatitlán were used as training sets, while model E+W+A+G was better when the training sets were Cd. Obregón and Zaragoza. Celaya and Cd. Obregón were always the best predicted sites. Compared to grain yield (Table 3), higher pairwise site predictions were observed for days to anthesis (Supplementary Table 3), days to maturity (Supplementary Table 4), and plant height (Supplementary Table 5). Accuracy of the prediction models’ values was higher than 0.54 for plant height and days to anthesis, whereas correlations ranged from 0.548 to 0.777 and from 0.613 to 0.749, respectively.

Grain yield predictions in untested environments (leave-one-out, Table 4) were performed using site information, environmental variables, pedigree, genotypic data, and pedigree by site and genomic by site interactions (E+W+A, E+W+A+AE, E+W+G+A, and E+W+G+A+GE+AE). Interestingly, leave-one-out accuracy overcomes pairwise-site accuracy indicating that four sites predict better one site than the pairwise-site comparison. Traits with higher heritability, as days to anthesis, maturity and plant height, were the ones best predicted by the leave-one-out (Supplementary Table 6). Among the seven tested models better results were obtained when predicting Celaya and Cd. Obregón for models E+W+G+A, E+W+A+AE, and E+W+G+A+GE+AE.

Average accuracy of including information from four sites (leave-one-site-out) increased from 0.66 to 0.76, 0.70 to 0.78, 0.41 to 0.57, and 0.27 to 0.36 for plant height, days to anthesis, days to maturity, and grain yield, respectively, relative to pair-wise comparisons (comparison of average values, Supplementary Tables 3–6, and Tables 3 and 4). Modelling the interactions in E+W+G+GE, and E+W+A+AE did not increase the prediction accuracy, whereas the main effect model E+W+G+E and the complete interaction model E+W+G+A+GE+AE increased the prediction of Celaya and Cd. Obregón for days to anthesis and maturity.

## Discussion

The identification of wheat genotypes with stable performance in diverse environments is a challenge for breeders, especially in countries where wheat can be grown in diverse agro-ecological zones with high soil diversity and various patterns of precipitation and temperature. In this study, performance of diverse wheat lines was screened at multiple sites, encouraging local breeders to evaluate diverse germplasm in their environments and with their best management practices. By growing the lines in different environments, we expected to include in predictive model the environmental factors influencing the yield ranking of cultivars from site to site. The trait and site analysis are the important pre-requisites for determining the performance of genotypes across environments. In this investigation, all tested sites were positively correlated, i.e. in the same area of the bi-plot (Fig. 3). Also, most of genotypes were grouped in the center of the bi-plot, indicating for their similar response across the sites.

As expected, screening ability was highest for sites with no major prevailing abiotic and biotic stresses^23^. Celaya and Cd. Obregón had the highest capacity for discriminating performance by genotype, and thus, ideal for the selection of superior lines. Cd. Obregón, a temperate high-radiation irrigated environment, and one of the CIMMYT’s principal test sites, has been identified as one of the most suitable environments for screening under optimal conditions and for simulating different environmental stresses (e.g. drought, heat). It was interesting to note that sites Celaya and Cd. Obregón which showed the maximum separation from origin, resembled temperature regimes during grain filling (Fig. 2). This contributes to a comparable heritability pattern in these two sites for traits days to maturity, days to flowering, and plant height.

Genomic predictions have been performed in wheat for agronomically relevant traits^24^ with aim to accelerate genetic gains. High quality predictions with high accuracy for genomic selection programs can be expected at the sites with the highest heritability (Celaya and Cd. Obregón). This is particularly important, considering that investments in high quality phenotyping are needed to fully utilize its potential to complement genome sequencing as a route to rapid advances in breeding. However, the increasing temperatures witnessed over the past decade have been identified as one of the limiting factors that significantly reduce wheat production in this area of Mexico (Celaya and Cd. Obregón). Lobell *et al.*^25^ reported 7–12% yield losses for northwest Mexico for each degree Celsius rise in temperature. An integrated approach combining the latest genomics resources with physiological research^26^ would be needed to understand complex quantitative traits like grain yield under the environmental constraints resulting from climate change. Environment descriptors are easily available nowadays, increasing the opportunities for using multiple sources of information and variables of different nature to improve the model. However, it is reasonable to use biologically relevant covariables, related to specific plant functions. In a nutshell, genomic selection for grain yield would be more effective for sites that are showing high heritability/repeatability and are less affected by biotic/abiotic stresses. Environmental variables can play an important role in determining success of the prediction models. In this study we report the first attempt to predict performance of genotypes in unobserved environments by modeling, thereby incorporating the environment effect in prediction.

Results showed that the prediction models that simultaneously included site (E), genomic and pedigree (G, A), and environmental data (W) consistently gave higher predictions for both CV1 and CV2, pairwise-site, and leave-one-site-out. This study indicates that accounting for environment data increases the predictive ability of the model using random cross-validation. This conclusion concurs with the findings of Jarquin *et al.*^13^ in wheat trials and of Crossa *et al.*^27^, where increases in prediction accuracies were achieved by including dense molecular markers and G × E in a set of Mexican and Iranian landraces. Our study therefore provides a proof of concept that incorporating environmental variables in prediction models enhances their power ultimately making them more suitable and practical for climate resilient wheat improvement. A systematic robust analysis involving wheat mega-environments (other than Mexico) will ensure a wide spread application of this comprehensive research approach.

Predicting the performance of lines that have never been evaluated in the field (CV1) was more challenging than predicting the performance of lines that were evaluated in different environments (CV2). In this study, prediction accuracy from CV2 was higher than those obtained in CV1, indicating the contribution of the information from correlated environments when predicting yield performance (Table 2). In addition to these prediction problems, this study evaluated the predictions for different traits in untested environments concluding that environments where no genotypes were previously evaluated can still be predicted with good accuracy. However, environmental covariables from the untested environments are required and positive correlation between environments is still an important factor for achieving good prediction accuracy of unobserved environments. In a recent article, Jarquin *et al.*^28^, optimized training sets for genomic prediction of soybean accessions using independent validation trials such as leave-one-site-out with no environmental covariables; the authors show high prediction accuracy for % protein and grain yield.

Overall, results suggested that efforts on genomic selection for grain yield must include interdisciplinary teams and collaborative projects, with cross-validation protocols helping to test the potential accuracy of predictions. Simultaneously, the selection of appropriate sites for screening germplasm need to be decided appropriately when applying genomic selection in germplasm enhancement programs for fast track-efficient-precision breeding.

## Materials and Methods

### Plant material

A set of 803 spring wheat lines (*Triticum aestivum* L.) was selected from various sources, including CIMMYT International Nurseries (elite germplasm) and the Generation Challenge Program spring wheat reference set, a panel including diverse accessions with potential for favorable allele mining.

### Climatic data

The study was conducted under optimal conditions at five different environments (i.e. five sites, Fig. 1) in Mexico during 2011–12. Map in Fig. 1 was constructed using ESRI’s ArcGIS Desktop ArcMap 10.2.2 software^27^,^28^,^29^. The list of sites, coordinates for each site (latitude, longitude, and altitude), wheat cycle data (sowing and harvesting date), and meteorological data from the nearest meteorological station (including average, maximum and minimum temperature) are shown in Supplementary Table 1. Sites covered a wide range of environmental conditions in Mexico: altitude ranged from 39 to 1930 masl and latitude ranged from 20–28 degrees N. The average temperature during the season was 19.7 °C; minimum temperature was 11.1 °C and maximum temperature was 28.7 °C.

Planting dates varied from Nov 2011 to Jan 2012. All trials were gown under fully irrigated conditions with adequate pest control. Manual and/or chemical weed control was also applied as required. Seeds were sown in two row plots of length 1.0 m and width 0.8 m, with 0.2 m between rows. Seeding rate was approximately 150 grams m^−2^. A partially replicated experimental design (p -rep) in augmented blocks was used, where 81% of the accessions were repeated once, 15% were repeated twice, 4% were repeated three or more times, and 6% of the plots were used with checks.

### Phenotypic trait evaluation

Measurements were taken according to the protocols detailed in Pask *et al.*^30^. Days to anthesis was recorded as the number of days from planting until >50% of the spikes in each plot had completely emerged above the flag leaves and flowering had begun in the middle of the head. Days to maturity was similarly recorded as the number of days from planting until 50% of the peduncles in each plot had turned yellow. Plant height was the distance from the soil surface to the tip of the spike (excluding awns), taken as the average of three values for each plot in the field. Grain yield was the total weight of seed in each plot, divided by the plot area and expressed as t ha^−1^.

### Genotypic characterization

Genomic DNA was extracted from fresh leaves using a modified cetyltrimethyl-ammonium bromide method^31^. DNA quality and concentration were determined by electrophoresis in 1% agarose gel. A high-throughput genotyping method using DArT-Seq^TM^ technology^32^,^33^ was employed to generate genomic profiles of the population presented in this study. A complexity reduction method including two enzymes (*PstI* and *HpaII*) was used to create a genome representation of the set of samples^32^,^33^,^34^. *PstI*-RE site specific adapter was tagged with 96 different barcodes enabling multiplexing a 96-well microtiter plate with equimolar amounts of amplification products in order to run within a single lane on Illumina HiSeq2500 instrument (Illumina Inc., San Diego, CA). The successfully amplified fragments were sequenced up to 77 bases, generating approximately 500,000 unique reads per sample. Thereafter the FASTQ files (full reads of 77 bp) were quality filtered using a Phred quality score of 30, which represent a 90% of base call accuracy for at least 50% of the bases. More stringent filtering was also performed on barcode sequences using a Phred quality score of 10, which represents 99.9% of base call accuracy for at least 75% of the bases. A proprietary analytical pipeline developed by DArT P/L was used to generate allele calls for SNP and presence/absence variation (PAV) markers. Then, a set of filtering parameter was applied to select high quality markers for this specific study. One of the most important parameters is the average reproducibility of markers in technical replicates for a subset of samples which was set at 99.5%. Another critical quality parameter is call rate. This is the percentage of targets that could be scored as ‘0’ or ‘1’, the threshold was set at 50%.

## Data analysis

### Analysis of phenotypic data and G × E interaction

Individual analysis of sites was performed using a mixed linear model in order to obtain the best linear unbiased prediction (BLUP) and trait heritability. Group effects were determined by the entries classified as checks versus accessions. Components of variance were also estimated. Days to heading, days to maturity, plant height, and grain yield were analyzed using a mixed linear model in five environments.

The linear mixed model for the combined analyses is:


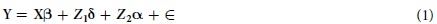


where ‘**Y**’ is the vector of response variable, ‘**X**’ is the incidence matrix of fixed effects (sites), ‘**β**’ is the vector of effects of environments, ‘**Z**_**1**_’ is the incidence matrix of random effects of block nested in sites, ‘**δ**’ is the vector of effects of blocks nested in sites, ‘**Z**_**2**_’ is the incidence matrix of random effects of genotype nested in sites, ‘**α**’ is the vector of effects of genotype by site interaction and ‘**∈**’ is the experimental error


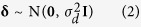



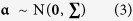



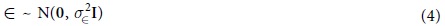



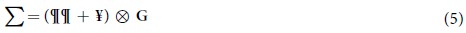


where ‘**¶**’ is the loading matrix of s (number of sites) rows by number of factors (2) columns, ‘**¥**’ is a diagonal matrix containing site specific variances and **G** is the matrix of relationships between genotypes obtained from the marker matrix. This model is known as the factor analytical model. It is able to model the environmental component of the G × E interaction in a suitable way to interpret it and borrowing information between correlated sites. Inclusion of the **G** matrix, produces more reliable results with lower standard error of the BLUPs. Three checks were included, their effects and the effects of the accessions were estimated separately as well as their interactions with the sites.

### Predictive Statistical Models

The models considered different combinations for the effects of lines (L), sites (E), genotypic data (G), pedigree (A), and environmental variables (W). Further details of the models outlined below can be found in Jarquin *et al.*^13^. We initially described the baseline model and then seven reaction norm models using pedigree and genomic relation matrices as well as environmental covariates.

### Baseline model

The phenotypic response variable (*y*_*ijk*_) is described as the sum of an overall mean (*μ*) plus random deviations due to the environment *E*_*i*_ (*i* = 1, …*I*) and the line *L*_*j*_ (*j* = 1, …*I*), plus an error term *ε*_*ijk*_(*k* = 1, …*r*_*ij*_). The linear mixed effects models is


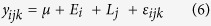


where 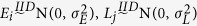 and 
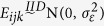
 and N(.,.) denotes a normal density and IID stands for independent and identically distributed.

### Model 1 (L+E+W)

Environmental co-variables (EC) are introduced in the baseline model. We add in equation [6] a random regression on the ECs (**W**) that describes the environmental conditions faced by each line in each environment, that is: 
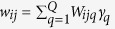
, where *W*_*ijq*_ is the value of the *q*^*th*^ EC evaluated in the *ij* environment × line combination, γ_*q*_ is the main effect of the corresponding EC, and *Q* is the total number of EC. We regarded the effects of the ECs as IID draws from normal densities, that is: 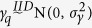 . Therefore, the vector *w* = *W*_*γ*_ follows a multivariate normal density with null mean and a covariance matrix proportional to Ω ∝ W*W′*. This covariance structure describes the similarity between environmental conditions.

Therefore, when the effects of the EC are added to equation [6] the model becomes


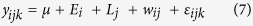


with 
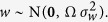


### Model 2 (E+W+G)

When markers are available we replace in equation [7] the random effect of the line (*L*_*j*_) with a regression on marker covariates of the form: 
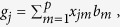
 where *g*_*j*_ represents an approximation of the true genetic value of the *j*^*th*^ line, *x*_*jm*_ is the genotype of the *j*^*th*^ line at the *m*^*th*^ marker, and *b*_*m*_ is the effect of the *m*^*th*^ marker. We regarded marker effects as IID draws from normal distributions of the form 
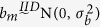
, (*m = *1, …*, p*).

The vector **g = Xb** containing the genomic values of all the lines follows a multivariate normal density with null mean and covariance-matrix 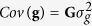, where **G** is a genomic relationship matrix whose entries are given by **G** = (**XX**)/(*p*)(Van Raden, 2008). Thus, we have the standard GBLUP model plus the random environmental effect (*E*_*i*_) and the effects of the EC (*w*_*ij*_):


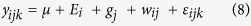


with 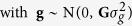.

Note that the effects of the level of the random effects ***g*** = (*g*_1_, .., *g*_*J*_) are correlated according to the off-diagonal values of **G**. There is thus the potential to borrow information across lines allowing, for example, prediction of the performance of lines that have not yet been evaluated in any field trial.

### Model 3 (E+W+G+GE)

Adding to model 2 the interaction between genomic (markers) and environments we developed model 3. Jarquín *et al.*^13^ showed that, under standard assumptions, the covariance structure induced by the reaction-norm model is the Hadamard (cell-by-cell) product of two (co)variance structures one describing the relationships between lines based on genetic information, e.g., **G,** and one describing environmental effects (*E*_*i*_). We extended the model in equation [8] by adding a new random effect representing interactions between the genomic and the environmental effects, **gE** such that 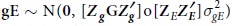 where  o stands for the Hadamard product and 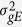 is the genomic × environment interaction parameter. Then the model becomes:


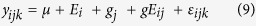


### Model 4 (E+W+A)

A modification of model 2 is to incorporate pedigree information using the additive relationship matrix **A** (*a*_*j*_). The model becomes:


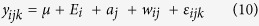


The vector **a** = (*a*_1_, .., *a*_*J*_) contains the additive random effect of the lines and it is assumed to have a normal distribution 
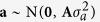
where 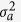
is an additive variance parameter.

### Model 5 (E+W+A+AE)

Similar to model 2 but incorporating the random interaction effects between the pedigree of the lines (*a*_*j*_) and the effect of the environments (*E*_*i*_), with **aE** such that 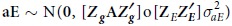 where o stands for the Hadamard product and 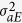 is the pedigree × environment interaction parameter. Then the model becomes:


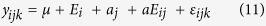


### Model 6 (E+W+G+A)

This random linear model has only main effects environments, C, genomic and pedigree.


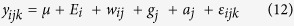


### Model 7 (E+W+G+A+GE+AE)

This model has the four main effects (*E*_*i*_, *w*_*ij*_, *g*_*j*_, and *a*_*j*_) and the two possible interactions (*gE*_*ij*_ and *aE*_*ij*_)


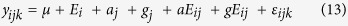


### Assessing model prediction accuracy of different prediction problems

Following Burgueño *et al.*^9^, we initially considered two distinct prediction problems by cross-validation 1 (CV1) and cross-validation 2 (CV2). Cross-validation CV1 measures the ability of models to predict the performance of a subset of lines that have not yet been evaluated in any of the environments included in the multi-environment trials. CV2 measured the ability of models to predict the performance of lines using data collected in sparse environments. In CV1 we randomly assigned lines to folds, thus ensuring that all the records of a given line were assigned to the same fold. In CV2 we randomly assigned individual plot records to folds; with this setting individual records of a given line are potentially assigned to different folds. The size of the training-testing sets for the two random cross-validations was of 80–20%. For CV1, 20% of the lines (around 160 wheat lines) were not observed in any of the 5 Mexican sites and for CV2, some of the 20% of the lines were observed in some sites but not in the others.

Another prediction problem studied was the direct prediction of one site using another site (pairwise-site) for all pair of sites. A new prediction problem was studied and denoted as leave-one-site-out; this was added to explain the ability of the model to predict the performance of wheat lines in environments that were not used in the training and where the only available information from them is the collected climatic data. The leave-one-site-out differed from the pairwise-site because four environments were used to predict another one.

## Additional Information

**How to cite this article**: Saint Pierre, C. *et al.* Genomic prediction models for grain yield of spring bread wheat in diverse agro-ecological zones. *Sci. Rep.* **6**, 27312; doi: 10.1038/srep27312 (2016).

## Supplementary Material

Supplementary Information

## Figures and Tables

**Table 1:** Genetic variances and correlations between sites for grain yield, plant height, days to anthesis and maturity.

Sites	Celaya	Delicias	Tepatitlán	Cd. Obregón	Zaragoza
Grain yield/Plant height					
Celaya	**3.43/147**	0.899	0.937	0.843	0.859
Delicias	0.514	**2.39/70**	0.849	0.837	0.930
Tepatitlán	0.735	0.389	**2.43/121**	0.857	0.827
Cd. Obregón	0.849	0.444	0.697	**3.29/93**	0.797
Zaragoza	0.832	0.446	0.794	0.829	**0.48/123**
Days to maturity/Days to anthesis					
Celaya	**46.5/37.5**	0.851	0.875	0.875	0.834
Delicias	0.876	**25.6/32.6**	0.894	0.949	0.928
Tepatitlán	0.874	0.838	**11.7/69.8**	0.952	0.898
Cd. Obregón	0.881	0.846	0.846	**68.7/74.9**	0.921
Zaragoza	0.750	0.729	0.738	0.994	**12.4/48.8**

For grain yield and plant height the diagonal shows the variance components (./.), the lower diagonal the correlation between sites for grain yield, and the upper diagonal the correlation between sites for plant height. For days to maturity-days to anthesis the diagonal shows the variance components (./.), the lower diagonal the correlation between sites for days to maturity and the upper diagonal the correlation between sites for days to anthesis.

**Table 2:** Average correlation (and standard deviation in parenthesis) between observed and predicted values for two Random cross-validation schemes for trait grain yield (YLD) in five sites in Mexico; L = line, E = site, G = genotypic information, A = pedigree, W = environmental variables, and interactions.

Site	Genomic model						
	L+E+W	E+W+G	E+W+G+GE	E+W+A	E+W+A+AE	E+W+G+A	E+W+G+A+GE+AE
CV1							
Celaya	−0.062	0.369	0.364	0.429	0.427	**0.432**	0.419
	(0.043)	(0.012)	(0.013)	(0.009)	(0.010)	(0.010)	(0.011)
Delicias	−0.065	0.130	0.131	0.190	0.189	0.189	0.199
	(0.035)	(0.011)	(0.023)	(0.010)	(0.017)	(0.009)	(0.019)
Tepatitlán	−0.046	0.260	0.291	0.299	0.258	**0.319**	0.311
	(0.044)	(0.008)	(0.012)	(0.007)	(0.015)	(0.007)	(0.014)
Cd. Obregón	−0.060	0.444	0.481	0.523	0.571	0.538	**0.581**
	(0.040)	(0.012)	(0.010)	(0.009)	(0.010)	(0.009)	(0.010)
Zaragoza	−0.020	0.198	0.228	0.221	0.209	0.238	**0.252**
	(0.035)	(0.009)	(0.015)	(0.009)	(0.018)	(0.007)	(0.016)
CV2							
Celaya	0.397	0.461	0.433	**0.491**	0.478	0.484	0.466
	(0.004)	(0.004)	(0.009)	(0.005)	(0.008)	(0.004)	(0.008)
Delicias	0.221	0.218	0.214	0.244	0.248	0.241	**0.249**
	(0.003)	(0.004)	(0.018)	(0.005)	(0.015)	(0.004)	(0.018)
Tepatitlán	0.324	0.352	0.384	0.350	0.318	0.364	**0.367**
	(0.003)	(0.005)	(0.010)	(0.005)	(0.013)	(0.004)	(0.012)
Cd. Obregón	0.423	0.517	0.536	0.563	0.604	0.558	**0.613**
	(0.007)	(0.007)	(0.009)	(0.006)	(0.006)	(0.006)	(0.006)
Zaragoza	0.270	0.279	0.303	0.271	0.267	**0.286**	0.282
	(0.001)	(0.004)	(0.007)	(0.004)	(0.008)	(0.003)	(0.008)

Cross-Validation CV1: predictions when a proportion of lines are not included in any of the 5 sites; Cross-Validation CV2: predictions when a proportion of lines are removed from some of the sites and left in others.

**Table 3:** Pair-wise correlation between the observed and predicted values for grain yield for four models; L = line, E = site, G = genotypic information, A = pedigree, W = environmental variables.

Genomic Model	Testing site	Training site				
		Celaya	Delicias	Tepatitlán	Cd. Obregón	Zaragoza
**L+E+W**	Celaya	—	0.159	0.267	0.376	0.148
	Delicias	0.157	—	0.126	0.194	0.103
	Tepatitlán	0.267	0.126	—	0.261	0.208
	Cd. Obregón	0.377	0.193	0.262	—	0.247
	Zaragoza	0.148	0.105	0.211	0.245	—
**E+W+G**	Celaya	—	0.273	0.346	0.422	0.288
	Delicias	0.183	—	0.164	0.166	0.136
	Tepatitlán	0.305	0.230	—	0.280	0.252
	Cd. Obregón	0.480	0.267	0.373	—	0.398
	Zaragoza	0.201	0.153	0.211	0.248	—
**E+W+A**	Celaya	—	0.293	0.404	0.450	0.295
	Delicias	0.209	—	0.255	0.176	0.190
	Tepatitlán	0.325	0.281	—	0.277	0.255
	Cd. Obregón	0.529	0.294	0.422	—	0.428
	Zaragoza	0.206	0.179	0.209	0.249	—
**E+W+G+A**	Celaya	—	0.286	0.377	0.449	0.310
	Delicias	0.203	—	0.203	0.180	0.174
	Tepatitlán	0.324	0.267	—	0.290	0.276
	Cd. Obregón	0.515	0.285	0.404	—	0.440
	Zaragoza	0.206	0.177	0.228	0.256	—

Values from one site (training site) were used to predict a second site (testing site).

**Table 4:** Correlation between the observed and predictive values of the leave-one-site-out prediction problem (prediction of one site when all the other sites are used in the model); L = line, E = site, G = genotypic information, A = pedigree, W = environmental variables and the interactions.

Sites	Genomic model						
	L+E+W	E+W+G	E+W+G+GE	E+W+A	E+W+A+AE	E+W+G+A	E+W+G+A+GE+AE
Celaya	0.403	0.459	0.440	0.488	0.466	0.480	0.435
Delicias	0.225	0.212	0.224	0.235	0.240	0.231	0.228
Tepatitlán	0.327	0.341	0.350	0.352	0.354	0.358	0.358
Cd. Obregón	0.432	0.487	0.438	0.513	0.497	0.511	0.516
Zaragoza	0.272	0.277	0.283	0.271	0.270	0.280	0.286
