# Supplementary material for: Genomic prediction models for grain yield of spring bread wheat in diverse agro-ecological zones
Source: Sci Rep. 2016 Jun 17;6:27312. doi: 10.1038/srep27312 (PMC4911553; doi:10.1038/srep27312)
Supplement: Supplementary Information [file srep27312-s1.pdf]

Genomic prediction models for grain yield of spring bread wheat in diverse agro-ecological zones

C. Saint Pierre[1 ¶], J. Burgueño[1 ¶], J. Crossa[1 ¶], G. Fuentes Dávila[2], P. Figueroa López[2], E. Solís Moya[2], J. Ireta Moreno[2], V. M. Hernández Muela[2], V. Zamora Villa[3], P. Vikram[1], K. Mathews[1], C. Sansaloni[1], D. Sehgal[1], D. Jarquin[4], P. Wenzl[1$] and Sukhwinder-Singh[1]*

[1]International Maize and Wheat Improvement Center (CIMMYT), Km. 45, Carretera México-Veracruz, El Batán, Texcoco, México, CP 56237

[2]Instituto Nacional de Investigaciones Forestales, Agrícolas y Pecuarias, INIFAP, México;

[3]Universidad Autónoma Agraria Antonio Narro, México;

[4]Department of Agronomy and Horticulture, University of Nebraska-Lincoln, 321 Keim Hall, Lincoln, NE, 68503-0915, USA

[$]Present address: Centro Internacional de Agricultura Tropical (CIAT), Km 17 Recta Cali-Palmira, 763537 Cali, Colombia

[¶] First three authors contributed equally to this work.

* Corresponding author:
Sukhwinder-Singh, Wheat Lead, Seeds of Discovery (SeeD) initiative

International Maize and Wheat Improvement Center (CIMMYT)

Km. 45, Carretera México-Veracruz, El Batán, Texcoco CP 56130

Edo. de México. MEXICO

E-mail: suk.singh@cgiar.org

Mexico City: +52 (55) 5804 2004

Texcoco, México: +52 (595) 952 1900; ext. 2104, Mobile: +52 1 595 1255378

Supplementary Table 1: List of sites (city and State in Mexico), coordinates (latitude, longitude and altitude), wheat cycle (sowing and harvesting date), and meteorological data including average, maximum and minimum temperature, for the environments where the wheat panel was sown.

Site	Coordinates	Wheat Cycle	Month	Temp average	Temp max	Temp Min
Celaya, Guanajuato	20 34 48 N 100 49 11 W 1,752 metres above sea level (masl)	Winter 29-Nov2011-May2012	Nov-11	17.53	25.14	10.29
			Dec-11	16.76	24.94	9.12
			Jan-12	15.84	23.17	8.91
			Feb-12	16.69	22.93	11.29
			Mar-12	20.19	27.73	12.59
			Apr-12	21.84	29.38	14.05
			May-12	23.40	30.85	16.35
Delicias, Chihuahua	28 23 51 N 105 30 44 W 1,166 masl	Winter 13-Jan2012-Jun2012	Jan-12	12.86	23.16	2.56
			Feb-12	13.24	22.18	4.29
			Mar-12	17.03	27.16	6.90
			Apr-12	23.17	32.93	13.40
			May-12	25.88	34.73	17.03
			Jun-12	29.38	37.93	20.83
Tepatitlán, Jalisco	20 31 422 N 102 51 62 W 1,930 masl	Winter 4-Jan2012-Junio2012	Jan-12	17.03	27.53	6.53
			Feb-12	17.91	26.41	9.41
			Mar-12	21.10	31.61	10.58
			Apr-12	22.01	33.15	10.87
			May-12	24.89	35.68	14.10
			Jun-12	24.78	34.42	15.13
Ciudad Obregón, Sonora	27 22 12 N 109 55 43 W 39 masl	Winter 1-Dec2011-Apr2012	Dec-11	15.50	24.40	6.60
			Jan-12	15.48	28.54	6.26
			Feb-12	16.70	25.70	7.70
			Mar-12	18.15	28.30	8.00
			Apr-12	21.40	31.70	11.10
Zaragoza, Coahuila	28 35 56 N 100 54 43 W 343 masl	Winter 7-Dec2011-May2012	Dec-11	NA	NA	NA
			Jan-12	NA	NA	NA
			Feb-12	16.02	23.9	10.39
			Mar-12	19.51	27.41	12.23
			Apr-12	23.5	31.15	15.42
			May-12	23.54	30.22	18.44

Source: CIMMYT, INIFAP, http://clicom-mex.cicese.mx

Supplementary Table 2: Average correlation (and standard deviation in parenthesis) between observed and predicted values for two random cross-validation schemes for traits days to anthesis and maturity, and plant height in five sites in Mexico; L=line, E=site, G=genotypic information, A=pedigree, W=environmental variables and interactions. Cross-Validation CV1: predictions when a proportion of lines are not included in any of the 5 sites; Cross-Validation CV2: predictions when a proportion of lines is removed from some of the sites though leave in others.

	Sites	Genomic Model						
		L+E+W	E+W+G	E+W+G+GE	E+W+A	E+W+A+AE	E+W+G+A	E+W+G+A+GE+AE
------------ Days to anthesis ------------								
CV1	Celaya	-0.026 (0.048)	0.577 (0.013)	0.588 (0.013)	0.540 (0.011)	0.552 (0.012)	0.622 (0.010)	0.638 (0.010)
	Delicias	-0.019 (0.046)	0.529 (0.012)	0.522 (0.014)	0.497 (0.009)	0.482 (0.013)	0.564 (0.008)	0.550 (0.012)
	Tepatitlán	-0.027 (0.050)	0.540 (0.014)	0.573 (0.017)	0.480 (0.012)	0.481 (0.015)	0.561 (0.010)	0.599 (0.013)
	Cd.Obregón	-0.021 (0.051)	0.483 (0.012)	0.488 (0.015)	0.493 (0.012)	0.498 (0.015)	0.548 (0.010)	0.547 (0.013)
	Zaragoza	-0.019 (0.048)	0.505 (0.013)	0.509 (0.014)	0.497 (0.012)	0.500 (0.015)	0.557 (0.012)	0.567 (0.012)
CV2	Celaya	0.820 (0.001)	0.824 (0.001)	0.820 (0.003)	0.824 (0.001)	0.819 (0.003)	0.830 (0.001)	0.831 (0.003)
	Delicias	0.771 (0.001)	0.778 (0.001)	0.770 (0.003)	0.776 (0.001)	0.769 (0.003)	0.780 (0.001)	0.770 (0.003)
	Tepatitlán	0.773 (0.001)	0.784 (0.001)	0.813 (0.003)	0.772 (0.001)	0.769 (0.003)	0.780 (0.001)	0.810 (0.003)
	Cd.Obregón	0.791 (0.001)	0.787 (0.001)	0.798 (0.003)	0.787 (0.001)	0.800 (0.003)	0.792 (0.001)	0.808 (0.004)
	Zaragoza	0.757 (0.001)	0.763 (0.001)	0.769 (0.003)	0.764 (0.001)	0.768 (0.003)	0.768 (0.001)	0.782 (0.003)
------------ Days to maturity ------------								

CV1	Celaya	-0.047<br>(0.044)	0.346<br>(0.014)	0.320<br>(0.017)	0.290<br>(0.014)	0.258<br>(0.017)	0.358<br>(0.013)	0.336<br>(0.016)
	Delicias	-0.036<br>(0.040)	0.524<br>(0.013)	0.562<br>(0.011)	0.535<br>(0.011)	0.567<br>(0.011)	0.606<br>(0.012)	0.635<br>(0.011)
	Tepatitlán	-0.016<br>(0.038)	0.092<br>(0.009)	0.088<br>(0.021)	0.086<br>(0.011)	0.093<br>(0.022)	0.090<br>(0.009)	0.098<br>(0.024)
	Cd.Obregón	-0.038<br>(0.041)	0.388<br>(0.017)	0.405<br>(0.022)	0.375<br>(0.012)	0.419<br>(0.014)	0.435<br>(0.014)	0.466<br>(0.017)
	Zaragoza	-0.018<br>(0.040)	0.423<br>(0.015)	0.453<br>(0.017)	0.384<br>(0.010)	0.369<br>(0.014)	0.457<br>(0.011)	0.481<br>(0.014)
CV2	Celaya	0.643<br>(0.002)	0.617<br>(0.003)	0.544<br>(0.013)	0.605<br>(0.003)	0.531<br>(0.011)	0.621<br>(0.003)	0.547<br>(0.013)
	Delicias	0.689<br>(0.001)	0.729<br>(0.002)	0.740<br>(0.006)	0.721<br>(0.002)	0.740<br>(0.005)	0.738<br>(0.002)	0.771<br>(0.005)
	Tepatitlán	0.183<br>(0.001)	0.183<br>(0.002)	0.183<br>(0.008)	0.178<br>(0.002)	0.182<br>(0.005)	0.178<br>(0.002)	0.187<br>(0.006)
	Cd.Obregón	0.653<br>(0.003	0.659<br>(0.003)	0.630<br>(0.013)	0.680<br>(0.003)	0.673<br>(0.007)	0.678<br>(0.003)	0.667<br>(0.010)
	Zaragoza	0.663<br>(0.001	0.674<br>(0.002)	0.688<br>(0.005)	0.670<br>(0.002)	0.661<br>(0.005)	0.677<br>(0.002)	0.690<br>(0.006)
---------- Plant height ----------								
CV1	Celaya	-0.017<br>(0.040)	0.431<br>(0.019)	0.409<br>(0.023)	0.414<br>(0.012)	0.404<br>(0.016)	0.459<br>(0.013)	0.439<br>(0.018)
	Delicias	-0.016<br>(0.042)	0.496<br>(0.017)	0.535<br>(0.016)	0.491<br>(0.011)	0.499<br>(0.012)	0.547<br>(0.012)	0.578<br>(0.012)
	Tepatitlán	-0.014<br>(0.049)	0.450<br>(0.023)	0.492<br>(0.027)	0.386<br>(0.014)	0.370<br>(0.017)	0.462<br>(0.015)	0.508<br>(0.019)
	Cd.Obregón	-0.019<br>(0.037)	0.470<br>(0.016)	0.500<br>(0.014)	0.476<br>(0.013)	0.498<br>(0.014)	0.523<br>(0.013)	0.552<br>(0.013)
	Zaragoza	-0.024<br>(0.043)	0.525<br>(0.018)	0.555<br>(0.016)	0.516<br>(0.012)	0.547<br>(0.012)	0.586<br>(0.014)	0.617<br>(0.012)
	Celaya	0.728	0.727	0.718	0.713	0.712	0.723	0.718

		(0.001)	(0.001)	(0.006)	(0.001)	(0.004)	(0.001)	(0.005)
	Delicias	0.789	0.792	0.821	0.786	0.793	0.794	0.822
		(0.001)	(0.001)	(0.003)	(0.001)	(0.002)	(0.001)	(0.003)
	Tepatitlán	0.691	0.704	0.741	0.684	0.678	0.695	0.733
CV2		(0.001)	(0.001)	(0.007)	(0.001)	(0.005)	(0.001)	(0.007)
	Cd.Obregón	0.808	0.804	0.817	0.810	0.811	0.812	0.826
		(0.001)	(0.001)	(0.004)	(0.001)	(0.003)	(0.001)	(0.004)
	Zaragoza	0.813	0.818	0.841	0.823	0.837	0.826	0.854
		(0.001)	(0.001)	(0.003)	(0.001)	(0.003)	(0.001)	(0.003)

Supplementary Table 3: Pair-wise correlation between the observed and predicted values for days to anthesis for four models; L=line, E=site, G=genotypic information, A=pedigree, W=environmental variables. Values from one site (training site) were used to predict a second site (testing site).

		Training site				
Genomic Model	Testing site	Celaya	Delicias	Tepatitlán	Cd.Obregón	Zaragoza
**L+E+W**	Celaya	---	0.684	0.725	0.713	0.715
	Delicias	0.684	---	0.661	0.683	0.678
	Tepatitlán	0.727	0.660	---	0.702	0.616
	Cd.Obregón	0.713	0.683	0.701	---	0.668
	Zaragoza	0.714	0.678	0.613	0.665	---
**E+W+G**	Celaya	---	0.709	0.738	0.736	0.740
	Delicias	0.705	---	0.673	0.719	0.707
	Tepatitlán	0.745	0.683	---	0.730	0.640
	Cd.Obregón	0.708	0.700	0.705	---	0.680
	Zaragoza	0.727	0.700	0.626	0.690	---
**E+W+A**	Celaya	---	0.718	0.749	0.731	0.747
	Delicias	0.691	---	0.684	0.713	0.708
	Tepatitlán	0.736	0.697	---	0.714	0.636
	Cd.Obregón	0.710	0.718	0.708	---	0.678
	Zaragoza	0.730	0.722	0.635	0.685	---
**E+W+G+A**	Celaya	---	0.712	0.741	0.738	0.741
	Delicias	0.695	---	0.674	0.717	0.703
	Tepatitlán	0.739	0.688	---	0.727	0.633
	Cd.Obregón	0.718	0.708	0.709	---	0.680
	Zaragoza	0.730	0.709	0.626	0.692	---

Supplementary Table 4: Pair-wise correlation between the observed and predicted values for days to maturity for four models; L=line, E=site, G=genotypic information, A=pedigree, W=environmental variables. Values from one site (training site) were used to predict a second site (testing site).

		Training site				
Genomic Model	Testing site	Celaya	Delicias	Tepatitlán	Cd.Obregón	Zaragoza
**L+E+W**	Celaya	---	0.579	0.180	0.534	0.526
	Delicias	0.578	---	0.132	0.592	0.590
	Tepatitlán	0.180	0.131	---	0.137	0.154
	Cd.Obregón	0.534	0.591	0.138	---	0.570
	Zaragoza	0.529	0.590	0.152	0.571	---
**E+W+G**	Celaya	---	0.562	0.203	0.531	0.528
	Delicias	0.653	---	0.180	0.615	0.630
	Tepatitlán	0.172	0.128	---	0.144	0.161
	Cd.Obregón	0.567	0.594	0.194	---	0.591
	Zaragoza	0.569	0.598	0.224	0.587	---
**E+W+A**	Celaya	---	0.549	0.162	0.521	0.530
	Delicias	0.639	---	0.180	0.596	0.632
	Tepatitlán	0.173	0.123	---	0.146	0.153
	Cd.Obregón	0.583	0.604	0.200	---	0.615
	Zaragoza	0.569	0.594	0.162	0.577	---
**E+W+G+A**	Celaya	---	0.576	0.195	0.529	0.536
	Delicias	0.648	---	0.182	0.599	0.628
	Tepatitlán	0.178	0.130	---	0.144	0.159
	Cd.Obregón	0.585	0.606	0.199	---	0.601
	Zaragoza	0.575	0.602	0.205	0.576	---

Supplementary Table 5: Pair-wise correlation between the observed and predicted values for plant height for four models; L=line, E=site, G=genotypic information, A=pedigree, W=environmental variables. Values from one site (training site) were used to predict a second site (testing site).

		Training site				
Genomic Model	Testing site	Celaya	Delicias	Tepatitlán	Cd.Obregón	Zaragoza
**L+E+W**	Celaya	---	0.622	0.607	0.659	0.654
	Delicias	0.624	---	0.552	0.758	0.764
	Tepatitlán	0.608	0.550	---	0.626	0.643
	Cd.Obregón	0.660	0.756	0.625	---	0.727
	Zaragoza	0.654	0.762	0.642	0.728	---
**E+W+G**	Celaya	---	0.629	0.607	0.665	0.652
	Delicias	0.654	---	0.554	0.764	0.760
	Tepatitlán	0.636	0.561	---	0.641	0.650
	Cd.Obregón	0.681	0.757	0.628	---	0.717
	Zaragoza	0.686	0.770	0.648	0.735	---
**E+W+A**	Celaya	---	0.614	0.596	0.651	0.637
	Delicias	0.651	---	0.553	0.755	0.762
	Tepatitlán	0.623	0.548	---	0.622	0.640
	Cd.Obregón	0.692	0.759	0.632	---	0.726
	Zaragoza	0.690	0.777	0.658	0.736	---
**E+W+G+A**	Celaya	---	0.626	0.608	0.661	0.652
	Delicias	0.656	---	0.554	0.763	0.769
	Tepatitlán	0.633	0.554	---	0.630	0.648
	Cd.Obregón	0.689	0.762	0.629	---	0.730
	Zaragoza	0.690	0.772	0.649	0.735	---

Supplementary Table 6: Correlation between the observed and predictive values of the leaving-one-site-out prediction problem (prediction of one site when all the other sites are used in the model). L=line, E=site, G=genotypic information, A=pedigree, W=environmental variables and the interactions.

	Genomic model						
Site	L+E+W	E+W+G	E+W+G+GE	E+W+A	E+W+A+AE	E+W+G+A	E+W+G+A+GE+AE
---------- Days to anthesis----------							
Celaya	0.821	0.823	0.821	0.822	0.825	0.828	0.825
Delicias	0.772	0.778	0.764	0.776	0.777	0.779	0.764
Tepatitlán	0.774	0.779	0.779	0.772	0.772	0.778	0.778
Cd.Obregón	0.792	0.785	0.787	0.781	0.786	0.788	0.796
Zaragoza	0.758	0.761	0.756	0.763	0.759	0.766	0.758
---------- Days to maturity----------							
Celaya	0.647	0.612	0.640	0.603	0.594	0.611	0.632
Delicias	0.691	0.720	0.697	0.710	0.699	0.725	0.708
Tepatitlán	0.183	0.183	0.181	0.179	0.183	0.178	0.187
Cd.Obregón	0.656	0.655	0.653	0.674	0.673	0.674	0.641
Zaragoza	0.665	0.67	0.664	0.669	0.663	0.676	0.657
---------- Plant height----------							
Celaya	0.730	0.727	0.728	0.712	0.717	0.723	0.724
Delicias	0.790	0.789	0.788	0.784	0.787	0.792	0.791
Tepatitlán	0.694	0.698	0.695	0.685	0.688	0.692	0.689
Cd.Obregón	0.809	0.800	0.805	0.805	0.807	0.808	0.808
Zaragoza	0.814	0.816	0.805	0.818	0.809	0.822	0.799
